# Effects of modified twin block appliance in growing Class II high angle cases: A cephalometric study

**DOI:** 10.12688/f1000research.109040.3

**Published:** 2024-04-23

**Authors:** Kanistika Jha, Manoj Adhikari

**Affiliations:** 1 Lecturer and Consultant Orthodontics, College of Medical Sciences, Bharatpur, Chitwan, 44207, Nepal; 2 Lecurer and Consultant, Oral and Maxillofacial Surgeon, Nepalese Army Institute of health sciences, College of medicine., Kathmandu, 44600, Nepal

**Keywords:** Class-II, high angel cases, vertical growers, twin block, modified twin block, cephalometry, digitization, pubertal growth phase

## Abstract

**Background:**

Class II malocclusions represent anteroposterior dysplasia usually resulting from mandibular retrusion. Along with a retropositioned mandible, it can be associated with either upward or backward jaw rotation. High angle cases are often associated with a short ramal height, steeper mandibular plane, and large gonial angle. Twin block is a commonly used myofunctional appliance that incorporates bite planes that direct the occlusal forces in a more favorable direction for correction of the retrognathic mandible. We aimed to evaluate skeletal, dental, and soft tissue changes following modified twin block appliance therapy in high-angle cases.

**Methods:**

A cephalometric study was performed on 15 growing (10-14 years) high angle (Frankfort mandibular angle 28-35°) Class II Division I malocclusion patients undergoing twin block therapy. Skeletal, dental, and soft tissue changes were evaluated by cephalometric analysis using Dolphin software.

**Results:**

Pre- and post-treatment changes in cephalograms were assessed by analysis of variance and paired t-test. Significant changes in the position of the mandible (angle between Sella-Nasion-Point B [SNB] increased by 3.9 degrees, P=0.02), Wits appraisal (decreased by 1.54 mm, P=0.04), maxillo-mandibular relationship (angle between Point A-Nasion-Point B [ANB] decreased by 3.74 degrees, P=0.02) were observed. Soft tissue changes like the nasolabial angle were also significant, increasing by 3.8 degrees (P=0.04) and lower lip relation to E-line (reduction in lower lip protrusion) by 2 mm (P=0.04). Vertical parameters showed non-significant changes, like the Frankfort mandibular angle (FMA) increased by 0.07 degrees, (P=0.67), the angle between Sella-Nasion and Gonion-Gnathion (SN-Go-Gn) increased by 0.33 degrees, (P=0.67), Y-axis increased by 0.2 degrees, (P=0.32). The upper incisor inclination decreased non-significantly from 115.27±1.33 to 113.42±1.65 degrees, (P=0.12) and lower incisor increased non-significantly from 100.13±2.23 to 101.80 ±1.37 degrees, (P=0.08).

**Conclusions:**

Modified twin block appliance can be used to successfully treat Class II Division I high angle cases with good vertical control.

## Introduction

Class II malocclusions represent anteroposterior dysplasia that may result from maxillary protrusion, mandibular retrusion, or a combination of both, but the most common cause is mandibular retrusion. ^1^ Along with a retropositioned mandible, it can be associated with either upward or backward jaw rotation. ^2^ High angle cases are often associated with a short ramal height, steeper mandibular plane, and large gonial angle and anterior attachment of masseter muscle relative to the occlusal plane. According to Pepicelli, the vertical growers have a flaccid and weak type of muscle pattern with reduced bite force along with a long facial pattern. ^3^ The basic treatment objective of Class II vertical dysplasia in growing children would alter the amount and direction of jaw growth. According to Graber, the basic objective of the functional appliance is to train the musculature to assist in optimal dentofacial development by eliminating abnormal muscle functions. ^4^ Anderson V activator, ^5^ high posterior bite blocks, ^6^ and magnetic appliances ^7^ had shown promising results in directing the growth of vertical dysplasia. Twin Block is one of the commonly used myofunctional appliances which incorporates the bite planes that direct the occlusal forces in a more favorable direction for correction of the retrognathic mandible. ^8^ Clark has also described a modified version of the twin block appliance for high-angle cases. ^9^ Therefore, this study aimed to evaluate the cephalometric changes with a modified twin block appliance in growing Class II high angle cases.

## Methods

### Subjects

A total of 15 growing children with skeletal Class II Division I malocclusion within the age group of 10 to14 years, and falling under cervical vertebral maturation index (CVMI) stage 3 and 4 with Frankfort Mandibular Plane angle (28-35 degrees) were enrolled.

### Materials and methods

Pretreatment diagnostic records were taken, analyzed and a treatment plan was established with a twin block appliance. The bite registration was taken 2 to 3 mm beyond the freeway space (average 7 mm in premolar region) and at 3-4 mm interincisal opening. The twin block was fabricated with an increased posterior bite block as per Clark’s protocol for vertical growers with heat-cured acrylic materials. The inclined plane was modified with a mean value of 65 degrees and the posterior bite plate was kept intact, covering up to the last erupted molars, and no trimming was done. A mid-palatal expansion screw was incorporated for expansion in the upper arch. A long labial bow (stainless steel 0.7 inch) in the upper arch and a delta clasp on upper first molars and lower first premolars were incorporated for retention. Incisal capping was done to prevent the lower incisor proclination.

All patients were followed up after 24 hours for any discomforts and then every four weeks interval (Clark’s protocol ^10^). The questionnaires regarding pain in the muscles of mastication and temporomandibular joint (TMJ) area, difficulties with the appliance, duration of wearing were recorded. The expansion schedule was started after 10 days and consisted of one turn twice a week. The duration of the appearance of pterygoid response was recorded.

The treatment with the twin block was divided into stages. During the six to eight months study period, most subjects completed their active phase with the achievement of Class I molar relationship. The sagittal correction was maintained with the twin block. In follow-up visits, the appliances were examined for loose fit and discomforts. No trimming was done and the vertical dimension of posterior blocks was kept intact up to the last erupted molars. All the patients adapted well and were able to follow the proper protocol of appliance wear.

### Cephalometry

Standardized lateral skull radiographs were taken for all patients at 0 months, 6 months and 9 months stages, with the Frankfort horizontal plane parallel to plane and teeth in occlusion. All lateral cephalograms were traced using Dolphin software (Dolphin Imaging 11.95) and analyzed for skeletal, dental, and soft tissue changes after twin block therapy. All cephalograms were traced by a single investigator and checked twice.

### Patient consent

Written informed consent was obtained from all patients.

## Results

**Figure 1.**
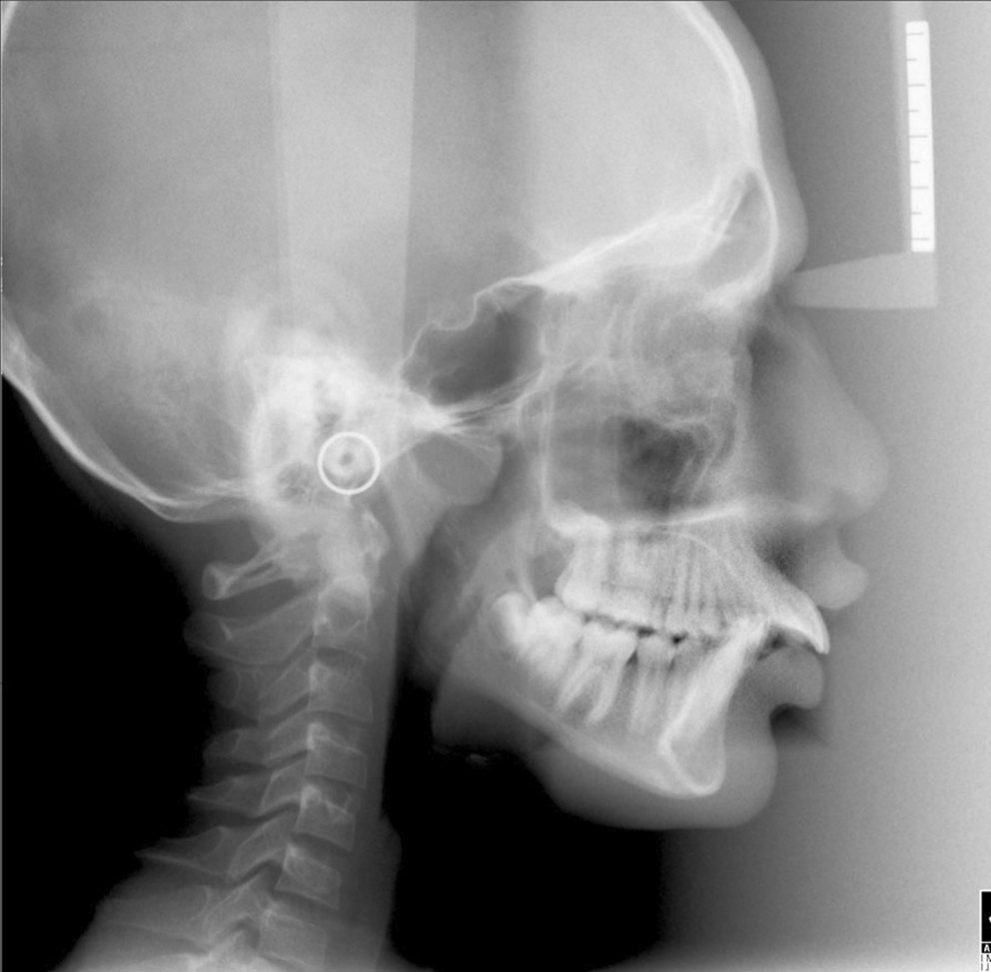
Pre-treatment cephalogram.

**Figure 2.**
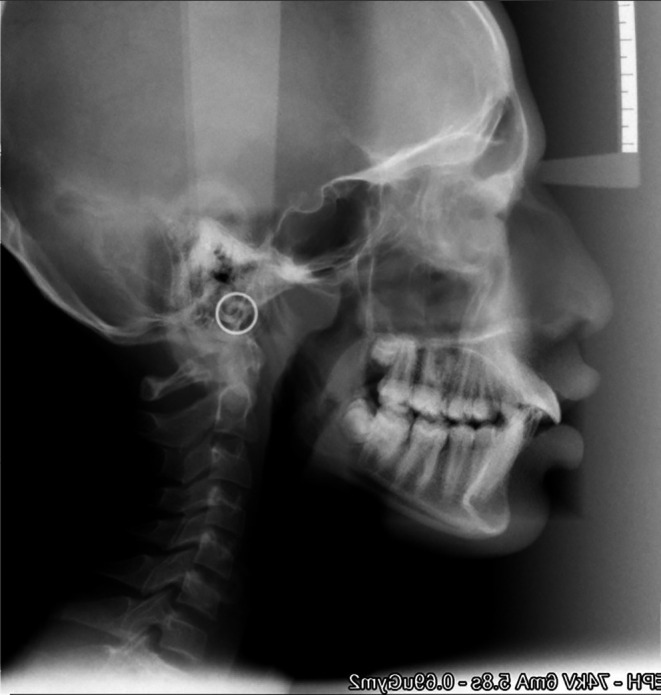
Post-treatment cephalogram.

**Figure 3.**
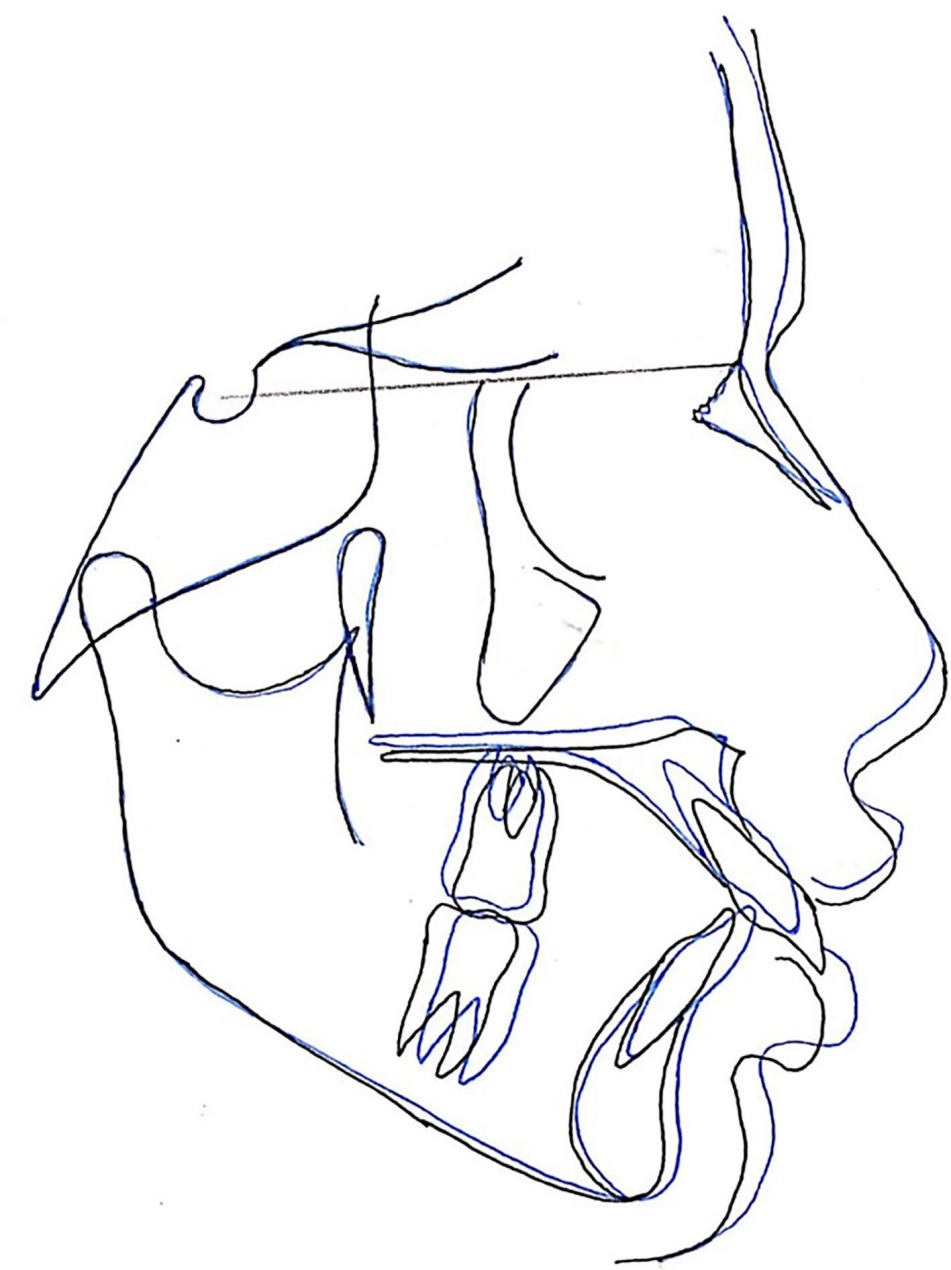
The superimposition of pre- and post-treatment average digitizations.

The mean age of the patients at the start of treatment was 12 years. Pre-and post-treatment lateral cephalograms were taken and evaluated for skeletal, dental, and soft tissue changes after wearing a twin block appliance therapy for nine months. Cephalometric changes during treatment are shown in Table 1. Figure 1 shows pre-treatment cephalograms, Figure 2 shows post-treatment cephalograms and Figure 3 shows the superimposition of pre-and post-treatment average digitizations. Twin block appliance wearing resulted in rapid skeletal correction as was evident from a statically significant decrease in angle Point A-Nasion-Point B (ANB), from 7.07±1.98 to 3.33±1.23 degrees (P=0.02); the angle of convexity decreased significantly from 9.87±1.5 to 5.13±1.24 degrees (P=0.01). There was a significant change in Wits appraisal, which decreased from 4.87±0.91 to 3.33±0.72 mm (P=0.04). Angle between Sella-Nasion-Point B (SNB) increased significantly from 74.0±1.46 to 77.9±0.77 degrees (P=0.02). There was a non-significant increase in mandibular length from 86.73±3.34 to 87.8±3.32 mm (P=0.1). The total anterior facial height (increased from 101.73±2.86 to 102.13±2.5 mm, P=0.09) and posterior facial height (increased from 57.27±1.53 to 57.40±1.68 mm, P=0.43) did not show significant changes with no significant increase in Frankfort mandibular angle (FMA increased from 30.73±1.48 to 30.80±1.37 degrees, P=0.67); there were no significant changes in Jarback ratio too (63.73±1.71 to 63.20±1.6, P=0.15); the angle between Sella-Nasion and Gonion-Gnathion (SN-Go-Gn) increased non-significantly from 132.27±2.43 to 132.60±2.29 degrees (P=0.67); the Y-axis increased non-significantly from 56.66±1.75 to 56.86±1.95 degrees (P=0.32). The upper incisor inclination decreased non-significantly from 115.27±1.33 to 113.42±1.65(P=0.12) and the lower incisor increased non-significantly from 100.13±2.23 to 101.80±1.37 degrees, (P=0.08). The nasolabial angle increased by 3.8 degrees and the change was statistically significant (P=0.04). The mean pretreatment (stage 0) value of overjet was 9.60±1.35 mm which was statistically significantly (P=0.01) reduced to 3.6±0.91 mm at 9 months (stage IV) of the twin block therapy. The mean overbite before the start of treatment (stage 0) was 6±0.92 mm. This changed to 2.47±0.83 mm at 9 months of twin block therapy. The change was statistically significant (P=0.03).

**Table 1.** Variable changes during treatment with TB appliances in Class II Division I vertical growers.

Variables	Pre-treatment (mean±SD)	Post-treatment (mean±SD)	P-value
SNA	81.33±1.135	80.6±1.143	0.1
SNB	74 .00± 1.46	77.90±0.77	0.02
ANB	7.07±1.98	3.33±1.23	0.02
Maxillary length	63.13±3.48	63.07±3.39	0.41
AO-BO (Wits appraisal)	4.87±0.91	3.33±0.72	0.04
Mandibular length	86.73± 3.34	87.80±3.32	0.10
N perpendicular to point A	1.67±0.81	1.60±0.63	0.67
N perpendicular to pogonion	7.07±1.03	4.47±0.91	0.03
FMA (degree)	30.73±1.48	30.80±1.37	0.67
SN-Go-Gn (degree)	132.27±2.43	132.60±2.29	0.67
Y-axis	56.66±1.75	56.86±1.95	0.32
Jaraback ratio	63.73±1.71	63.20±1.6	0.15
Total anterior facial height (mm)	101.73±2.86	102.13±2.5	0.09
Total posterior facial height (mm)	57.27±1.53	57.40±1.68	0.43
Upper incisor inclination (U1 to SN -degree)	115.27±1.33	113.42±1.65	0.12
Lower incisor angle (degree)	100.13±2.23	101.80±1.37	0.08
Angle of convexity (degree)	9.87±1.50	5.13±1.24	0.01
Nasolabial angle (degree)	99.87±2.56	103.67±2.19	0.04
Upper lip to E line (mm)	1.65±1.28	0.31±1.83	0.56
Lower lip to E line (mm)	2.92±1.34	0.92±1.27	0.04
Overjet (mm)	9.60±1.35	3.60±0.91	0.01
Overbite (mm)	6±0.92	2.47±0.83	0.03

Abbreviations: TB: twin block, SD: Standard deviation, SNA: angle between Sella-Nasion-Point A, SNB: angle between Sella-Nasion-Point B, ANB: angle between Point A-Nasion-Point B, AO-BO: distance between Point A-Occlusion line and Point B Occlusion line, FMA: Frankfort mandibular plane angle, SN-Go-Gn: angle between Sella-Nasion and Gonion-Gnathion line, NA: Line joining Nasion and point A, E line: Esthetic line.

## Discussion

The study was designed to evaluate the skeletal, dental, and soft tissue changes with a modified twin block appliance in high-angle cases. Growing children of ages 10-14 years in the cervical vertebra maturation index (CVMI) stages 3 and 4 were enrolled. Baccetti and McNamara stated that CVMI stages 3 and 4 represent the optimal treatment timing in dentofacial orthopedics. ^11^ Twin block appliances are patient-friendly, comfortable, designed to be worn for 24-hour periods. ^12^ The increased anterior facial height and high mandibular plane angle in vertical growers, and their response to the twin block appliance were determined in this study. The pre- and post-lateral cephalograms were digitized, and cephalometric parameters were determined with Dolphin software and rechecked manually twice. The main cephalometric parameter for correction of Class II was a significant increase in SNB angle, with insignificant change in the SNA angle indicating no maxillary skeletal change as suggested by Lund and Sandler. ^13^ A slight change in A point post twin block therapy is due to the remodeling of the bone of the anterior maxilla during retraction of upper anterior teeth. ^14^ The appliance was effective in correcting the Class II skeletal discrepancy with change in position and length of the mandible during the pubertal growth spurt. ^15^ Change in overjet and molar correction was due to a combination of skeletal and dentoalveolar changes with predominant skeletal changes. ^16^ The patients were treated during the peak growth spurt, which favored more skeletal changes than dental changes. Dentoalveolar correction was due to proclination of lower incisors and upper incisor retroclination. IMPA increased despite acrylic capping of the lower incisors. Upper incisor inclination was corrected with upper lip musculature force during functional treatment ^17^ and upper labial bow effects. ^13^^,^^15^ The high-bite twin block showed vertical control with no change in any vertical facial relationships, mandibular plane angle (FMA), and Jarabak ratio. Posterior bite blocks were kept intact without trimming. ^18^ Vertical control was due to having the acrylic block in the posterior region, which provides disocclusion of the teeth, removing the dental intercuspation, and releasing the mandibular growth, thus improving the Class II relationship. ^19^ So, the vertical dentoalveolar development could be controlled without changing the inclination of the mandibular plane as well as provide an additional increment of mandibular growth to the Class II correction. ^20^ The ratio of total anterior facial height to lower facial height remained the same pre- and post-functional treatment.

## Conclusion

Class II malocclusion in vertical growers is corrected by proclination of lower incisors, retroclination of upper incisors, distal movement of upper molars and/or mesial movement of lower molars, increase in mandibular length, and/or forward movement of the mandible. No significant changes on maxilla or mandible lengthening with good **v**ertical control can be done with a high-bite twin block appliance.

## Data availability

### Underlying data

Figshare: Effects of modified twin block appliance in growing Class II high angle cases: A cephalometric study, https://doi.org/10.6084/m9.figshare.19146296.v1. ^21^

This project contains the following underlying data:

- DATA excel sheet.xlsx

Data are available under the terms of the Creative Commons Attribution 4.0 International license (CC-BY 4.0).
