# Supplementary material for: Effects of modified twin block appliance in growing Class II high angle cases: A cephalometric study
Source: F1000Res. 2024 Apr 23;11:459. Originally published 2022 Apr 25. [Version 3] doi: 10.12688/f1000research.109040.3 (PMC11053348; doi:10.12688/f1000research.109040.3)
Supplement: Peer review history

# Reviewer report: Reviewer response for version 3

**DOI:** 10.5256/f1000research.165205.r267614

**Published:** 2024-05-08

**Authors:** Rudra Deo kumar

**Affiliations:** 1 All India Institute of Medical Sciences,, Patna, Bihar, India

No further comments to make.

Is the work clearly and accurately presented and does it cite the current literature?

Yes

If applicable, is the statistical analysis and its interpretation appropriate?

Yes

Are all the source data underlying the results available to ensure full reproducibility?

Yes

Is the study design appropriate and is the work technically sound?

Yes

Are the conclusions drawn adequately supported by the results?

Yes

Are sufficient details of methods and analysis provided to allow replication by others?

Yes

Reviewer Expertise:

Dentistry, Maxillofacial developmental anomaly, maxillofacial Trauma, Maxillofacial Pathology, Orthognathic Surgery etc.

I confirm that I have read this submission and believe that I have an appropriate level of expertise to confirm that it is of an acceptable scientific standard.

# Reviewer report: Reviewer response for version 3

**DOI:** 10.5256/f1000research.165205.r268994

**Published:** 2024-05-03

**Authors:** Alka Gupta

**Affiliations:** 1 Kantipur Dental college and Hospital, Kathmandu, Nepal

No further comments.

 Thank you

Is the work clearly and accurately presented and does it cite the current literature?

Yes

If applicable, is the statistical analysis and its interpretation appropriate?

Yes

Are all the source data underlying the results available to ensure full reproducibility?

Yes

Is the study design appropriate and is the work technically sound?

Yes

Are the conclusions drawn adequately supported by the results?

Yes

Are sufficient details of methods and analysis provided to allow replication by others?

Yes

Reviewer Expertise:

Orthodontics

I confirm that I have read this submission and believe that I have an appropriate level of expertise to confirm that it is of an acceptable scientific standard.

# Reviewer report: Reviewer response for version 2

**DOI:** 10.5256/f1000research.137346.r225537

**Published:** 2024-04-26

**Authors:** Rudra Deo kumar

**Affiliations:** 1 All India Institute of Medical Sciences,, Patna, Bihar, India

The current research paper demonstrates potential for indexing. It focuses on the adaptability of the Twin Block appliance, commonly utilized for horizontal jaw growth correction, to effectively address vertical growth patterns when modified accordingly. Emphasizing the necessity of early intervention using myofunctional appliances in treating skeletal malocclusion during a patient's developmental phase, the study underscores the importance of averting extensive Orthognathic surgery later in life.

 Notably, this research encourages clinicians to consider the modified Twin Block appliance as an effective approach for mandibular advancement in high-angle cases while maintaining excellent vertical control. Through comprehensive analysis, significant changes in various anatomical landmarks have been observed, supporting the efficacy of this modified appliance. Consequently, this study serves as a valuable resource for clinicians, offering insights into optimizing the use of myofunctional appliances.

 The paper's presentation is clear and precise, with thorough citations from current literature. Its robust study design and technically sound methodology make it replicable for other researchers. Moreover, the availability of the source data ensures complete reproducibility of the results. The conclusions drawn are well-founded, and adequately supported by the obtained results, contributing substantively to the field.

Is the work clearly and accurately presented and does it cite the current literature?

Yes

If applicable, is the statistical analysis and its interpretation appropriate?

Yes

Are all the source data underlying the results available to ensure full reproducibility?

Yes

Is the study design appropriate and is the work technically sound?

Yes

Are the conclusions drawn adequately supported by the results?

Yes

Are sufficient details of methods and analysis provided to allow replication by others?

Yes

Reviewer Expertise:

Dentistry, Maxillofacial developmental anomaly, maxillofacial Trauma, Maxillofacial Pathology, Orthognathic Surgery etc.

I confirm that I have read this submission and believe that I have an appropriate level of expertise to confirm that it is of an acceptable scientific standard.

# Reviewer report: Reviewer response for version 2

**DOI:** 10.5256/f1000research.137346.r238486

**Published:** 2024-02-19

**Authors:** Ankit Kumar Shahi

**Affiliations:** 1 ITS Centre for Dental Studies and Research, Ghaziabad, Uttar Pradesh, India; 2 Department of Orthodontics, ESIC Hospital (Ringgold ID: 81605), Delhi, Delhi, India

I would like to appreciate the author for a commendable work on this article on detailed view over the effects of modified Twin Block on Class II cases.

 However, there are few questions for better understanding: -

 1. What was the rationale for selecting the sample size of 15? and what was the number of males and females in the selected sample?

 2. The age group mentioned in abstract is 9-14, However in discussion section its mentioned 8-14 ...Kindly verify if there is any typing error. 

 3. Please elaborate about the significant changes in the Nasolabial angle as mentioned in the results and also please support it by any literature evidence 

 4. The article mentions in results and discussion about the good vertical control, however the superimposition provided is showing an increase in the anterior lower facial height with increase in vertical angle.

 Thanks, and regards.

Is the work clearly and accurately presented and does it cite the current literature?

Yes

If applicable, is the statistical analysis and its interpretation appropriate?

Partly

Are all the source data underlying the results available to ensure full reproducibility?

Yes

Is the study design appropriate and is the work technically sound?

Partly

Are the conclusions drawn adequately supported by the results?

Yes

Are sufficient details of methods and analysis provided to allow replication by others?

Yes

Reviewer Expertise:

Treatment of Class II malocclusion

I confirm that I have read this submission and believe that I have an appropriate level of expertise to confirm that it is of an acceptable scientific standard, however I have significant reservations, as outlined above.

# Response

**Published:** 2024-04-18

**Authors:** Manoj Adhikari

**Affiliations:** Nepalese Army Institute of Health Sciences, Nepal

1. What was the rationale for selecting the sample size of 15? and what was the number of males and females in the selected sample?

**Author response** : This is a pilot study so a sample size was taken as 15. Typically, the twin block appliance is contraindicated in cases with a high-angle presentation. The primary objective of this study is to determine the effectiveness of the twin block appliance in high-angle cases and to assess its impact on skeletal, dental, and soft tissue parameters. Gender is not considered as a variable in this study; however, there were 8 male and 7 female participants.

 2. The age group mentioned in abstract is 9-14, However in discussion section its mentioned 8-14 ...Kindly verify if there is any typing error.

**Author response: **The age group included in this study ranges from 10 to 14 years. A typing error in the discussion section has been rectified in version 3 of the article.

 3. Please elaborate about the significant changes in the Nasolabial angle as mentioned in the results and also please support it by any literature evidence

**Author response: ** The nasolabial angle shows a significant increase in this study, attributed to the retroclination of the upper incisors and the retrusion of the upper lip. Supporting evidence from the literature is provided below.

 “Khoja A, Fida M, Shaikh A. Cephalometric evaluation of the effects of the Twin Block appliance in subjects with Class II, Division 1 malocclusion amongst different cervical vertebral maturation stages. Dental press journal of orthodontics. 2016 May;21:73-84.”

 4. The article mentions in results and discussion about the good vertical control, however the superimposition provided is showing an increase in the anterior lower facial height with increase in vertical angle.

**Author response:** The twin block appliance exerts both horizontal and vertical effects. Vertical dimensional changes primarily result from appliance trimming; however, the blocks remain intact in this study. Therefore, vertical dimension changes may be attributed to mandibular growth rather than appliance action. Additionally, Figures 1, 2, and 3 have been updated in version 3.

# Reviewer report: Reviewer response for version 2

**DOI:** 10.5256/f1000research.137346.r225540

**Published:** 2023-12-18

**Authors:** Alka Gupta

**Affiliations:** 1 Kantipur Dental college and Hospital, Kathmandu, Nepal

The study's presentation is clear and scientific. The aim to treat Class II division 1 high angle cases using modified twin block appliance has been meet and successfully treated. The availability of the data ensures complete reproducibility of the results. However, the sample size calculation could be done more scientifically. This will provide a baseline data for the Class II division 1 high angle cases treated by Modified twin block.

Is the work clearly and accurately presented and does it cite the current literature?

Yes

If applicable, is the statistical analysis and its interpretation appropriate?

Yes

Are all the source data underlying the results available to ensure full reproducibility?

Yes

Is the study design appropriate and is the work technically sound?

Yes

Are the conclusions drawn adequately supported by the results?

Yes

Are sufficient details of methods and analysis provided to allow replication by others?

Yes

Reviewer Expertise:

Orthodontics

I confirm that I have read this submission and believe that I have an appropriate level of expertise to confirm that it is of an acceptable scientific standard.
